# Misrepair in Context: TGFβ Regulation of DNA Repair

**DOI:** 10.3389/fonc.2019.00799

**Published:** 2019-08-27

**Authors:** Qi Liu, Kirsten Lopez, John Murnane, Timothy Humphrey, Mary Helen Barcellos-Hoff

**Affiliations:** ^1^Department of Radiation Oncology, Helen Diller Family Comprehensive Cancer Center, University of California, San Francisco, San Francisco, CA, United States; ^2^Institute of Biomedical Engineering, Peking University Shenzhen Graduate School, Shenzhen, China; ^3^Shenzhen Bay Laboratory (SZBL), Shenzhen, China; ^4^Department of Oncology, CRUK/MRC Oxford Institute for Radiation Oncology, University of Oxford, Oxford, United Kingdom

**Keywords:** cancer, TGFβ, DNA repair, tumor microenvironment, genomic integrity, cytotoxic therapy, therapeutic response

## Abstract

Repair of DNA damage protects genomic integrity, which is key to tissue functional integrity. In cancer, the type and fidelity of DNA damage response is the fundamental basis for clinical response to cytotoxic therapy. Here we consider the contribution of transforming growth factor-beta (TGFβ), a ubiquitous, pleotropic cytokine that is abundant in the tumor microenvironment, to therapeutic response. The action of TGFβ is best illustrated in head and neck squamous cell carcinoma (HNSCC). Survival of HNSCC patients with human papilloma virus (HPV) positive cancer is more than double compared to those with HPV-negative HNSCC. Notably, HPV infection profoundly impairs TGFβ signaling. HPV blockade of TGFβ signaling, or pharmaceutical TGFβ inhibition that phenocopies HPV infection, shifts cancer cells from error-free homologous-recombination DNA double-strand-break (DSB) repair to error-prone alternative end-joining (altEJ). Cells using altEJ are more sensitive to standard of care radiotherapy and cisplatin, and are sensitized to PARP inhibitors. Hence, HPV-positive HNSCC is an experiment of nature that provides a strong rationale for the use of TGFβ inhibitors for optimal therapeutic combinations that improve patient outcome.

## Introduction

DNA repair is executed by multiple pathways that must be coordinated to deal with different types of DNA damage, including oxidative damage, single strand breaks (SSB), and double strand breaks (DSB). Complex intracellular mechanisms have developed to ensure an appropriate DNA damage response (DDR). In cancer, gene mutations and altered cell signaling can give rise to dysregulated and aberrant DNA repair mechanisms that presumably contribute to genomic instability and mutational burden that are associated with cancer progression.

Cancer cells are actively involved in crosstalk with host cells of the tumor microenvironment (TME), which includes the vasculature, immune cells and stroma, constitutes a robust but skewed signaling network distinct from normal tissue. Though the TME is critical in shaping the biology of a tumor, the impact of context-related signaling on the tumor cell's DNA repair proficiency is poorly understood. This article reviews cell intrinsic execution of DSB repair proficiency and pathway competency to discuss DNA repair in the context of the TME.

We focus on transforming growth factor-beta (TGFβ), which is critically involved in extrinsic control of pathway competency in DSB repair. Recently, we determined that compromised TGFβ signaling caused by human papilloma virus (HPV) in head and neck squamous cell carcinoma (HNSCC) shifts DSB repair to error prone and inefficient alternative end-joining (altEJ). HPV is an experiment of nature that provides compelling evidence that signaling from TGFβ, and thus the TME, is critical for DNA repair execution and pathway choice. The insight gained from understanding of the mechanisms by which TGFβ signaling, DDR, and TME are functionally linked, paves the way to further exploit weakness in specific cancers and develop pertinent therapeutic strategies.

## DSB Repair Pathways

**Figure 1:**
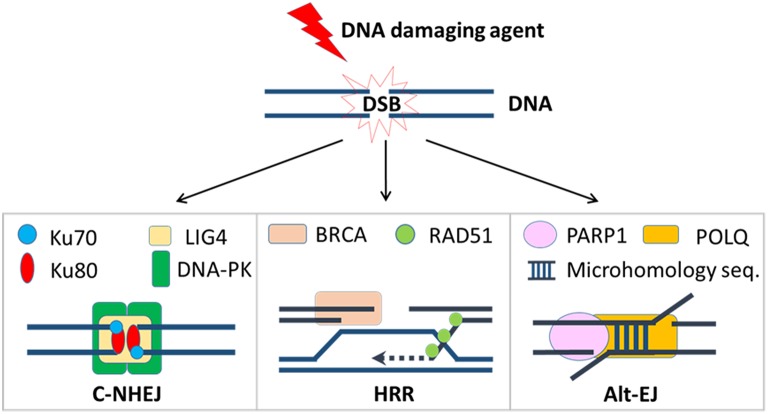
DSB repair pathways. Once a DSB is induced by a DNA damaging agent, cancer cells will try to repair it by one of these three mechanisms. Critical repair proteins in each pathway are shown.

Tens of thousands of DNA lesions are produced in a cell's daily life as a result of endogenous metabolic activities such as DNA replication or reactive oxygen species (ROS), as well as exposure to exogenous agents like ultraviolet (UV) or ionizing radiation (1, 2). Of the many types of DNA damage, DSBs are among the most dangerous. Failure to repair DSBs may lead to mutations, genomic, and chromosomal rearrangements, or cell death. In order to maintain genomic integrity, two predominant DSB repair pathways have developed to deal with different types of lesions: classical non-homologous end-joining (c-NHEJ) and homologous recombination repair (HRR) [Figure 1; (3)]. In mammalian cells, c-NHEJ is the predominant DSB repair pathway that can efficiently rejoin most DSBs (4). Although c-NHEJ functions throughout the cell cycle, it is particularly critical in the G1 phase when the cell has yet to replicate its DNA (5, 6). C-NHEJ is initiated by the binding of the Ku70/Ku80 heterodimer to the ends of the DSB, which is then recognized with high affinity by the catalytic subunit of DNA-dependent protein kinase (DNA-PKcs), forming the DNA-PK complex (7). This enables the recruitment of nucleases, including Artemis, to trim any short overhangs that are present on the DSB ends (8), and polymerases, including Polμ, to fill in any gaps (9–11). The final ligation step involves a ligase complex comprised of DNA ligase IV, X-ray repair cross-complementing protein 4 (XRCC4), and XRCC4-like factor (XLF), which is responsible for bridging and ligating the two processed ends (12). Because DSB ends often require processing to remove damaged nucleotides to enable ligation, c-NHEJ is considered an error-prone form of repair that can lead to short insertions and/or deletions. However, growing evidence suggests the context under which c-NHEJ is used in DSB repair is a critical determinant of the repair outcome, i.e., error-prone or error-prevention (13, 14). Although it awaits further evidence, it has been speculated that orchestrated repair with c-NHEJ in normal cells prevents chromosome instability, but in cancer cells with dysregulated repair pathways, inappropriate implementation of c-NHEJ-dependent end joining of non-contiguous ends can cause genomic alterations and lead to chromosome instability (13, 14).

The repair of DSBs by HRR is a highly complex process that requires the generation of single-stranded 3′ overhangs at each end, after which the homologous sequence on the sister chromatid is used to accurately fill in the gaps and restore the original DNA sequence (15, 16). In the first step of HRR, the DSB ends are recognized by the MRN complex, which is composed of meiotic recombination 11 homolog A (MRE11), RAD50, and Nijmegen breakage syndrome 1 (NBS1). Once bound to the DSB, the MRN complex activates ataxia telangiectasia mutated (ATM), a serine-threonine kinase that initiates the DDR by phosphorylating a plethora of substrates, thus facilitating the recognition of the DSB and the activation of downstream repair factors. In addition to its role in the MRN complex, MRE11 also associates with C-terminal binding protein interacting protein (CtIP), an endonuclease that allows for the removal of ~100 nucleotides from the 3′ end of the DSB (17, 18).

Following the initial processing step by MRE11 and CtIP, more extensive resection is subsequently mediated by either the EXO1 exonuclease or a combination of the RECQ helicase (BLM or RECQL4) and the DNA2 exonuclease (19). The single-stranded DNA formed by these resection steps is very quickly coated with and stabilized by replication protein A (RPA), preventing them from being degraded or forming DNA hairpins (20). For the final phase of HRR, RPA is replaced with RAD51 to facilitate homology search, strand invasion into the sister chromatid, and initiation of DNA synthesis at the region of the DSB (21). This process requires several proteins known as recombination mediators, e.g., the tumor suppressor breast cancer 1, early onset 2 (BRCA2) (22, 23). Repair is then completed upon resolution of crossover junctions by resolvases (24). Because HRR requires a homologous template, it can only be used to repair DNA that has been replicated (i.e., during the S and G2 phases of the cell cycle).

A third mechanism for repair of DSBs is altEJ, which is also referred to as backup end joining, or microhomology-mediated end joining (25, 26). A comparison of repair strategies is depicted in Figure 1. AltEJ uses poly [ADP-ribose] polymerase 1 (PARP1) to tether broken DNA ends (27), DNA polymerase theta (Pol θ) coded by the *POLQ* gene to initiate DNA replication at sites within two single-stranded 3′ overhangs (28), and DNA ligase I (LIG1) or DNA ligase III (LIG3) to join the DNA ends (29). AltEJ commonly occurs at sites containing short complementary sequences, known as microhomology, that are exposed after end resection; this requirement for resection and minimal homology means that altEJ has low fidelity and therefore frequently results in small deletions, insertions, and gross chromosomal rearrangements (30, 31). Because its execution increases genomic instability, altEJ is believed to be more active in certain cancers (32).

Other DSB repair pathways, such as single-strand annealing (SSA), can result in large deletions during repair by annealing of longer (e.g., >100 nt) repeats following extensive end-resection. These are rarely used in mammalian cells and have been reviewed recently (24), and will not be discussed herein.

## DSB Repair Pathway Competency in Cancer

The mechanism by which DSB are repaired is determined by a variety of factors, although the outcome is ultimately determined by the presence or absence of end resection. The initial phase of c-NHEJ, i.e., binding of the Ku heterodimer to DSB ends, minimizes end resection to allow accurate end-joining. End processing and resection are therefore tightly regulated by Ku70/Ku80, along with WRN and 53BP1, which together protect DNA ends during the G1 phase when HRR cannot occur due to the absence of a sister chromatid. Resection is also normally limited to late S or G2 due to the cell-cycle dependent expression of CtIP and its activation by CDK1 or CDK2 (33, 34). Importantly, resection requires the repositioning of 53BP1 on DSB ends by BRCA1, and therefore the loss of BRCA1 inhibits HRR, which was demonstrated by the fact that a deficiency in 53BP1 rescues the defect in HRR caused by the absence of BRCA1 (35). Noordermeer et al. recently demonstrated that 53BP1 effector complex, shieldin, localizes to DSB to prioritize c-NHEJ repair (36). In BRCA1-deficient cells, loss of shieldin or its subunits can restore HRR and resistance to PARP inhibition (37).

AltEJ was initially believed to be a backup pathway for c-NHEJ and HRR (26). The Ku heterodimer has much higher affinity for DSB ends relative to PARP1; thus, c-NHEJ is highly favored over altEJ in most circumstances (38). A higher frequency of altEJ-mediated repair was observed after the depletion of HRR factors such as RPA, BRCA1, and BRCA2 (39), suggesting HRR is used with priority in normal settings. In addition, because both HRR and altEJ require an initial resection step at DSB ends, both pathways are inhibited by c-NHEJ factors. Conversely, end resection is sufficient to block repair by c-NHEJ, as Ku70/Ku80 has very low affinity for single stranded DNA (40). Notably, accumulating evidence suggests that altEJ also competes with HRR for the repair of DSB (28, 41). For example, by studying dysfunctional telomeres and accumulation of RAD51 at DSBs, Mateos-Gomez and co-authors found that the loss of a critical component in altEJ, Pol θ, increased HRR in mice (28). Similar findings have been reported in ovarian cancers: HRR was upregulated when Pol θ expression was inhibited, while Pol θ expression blocks RAD51-mediated HRR due to RAD51 binding motifs in Pol θ (41).

Cell cycle phase plays an important role in DSB repair pathway choice. In S and G2 phases, HRR is preferentially used to repair DSB due to the presence of CYREN, an inhibitor of c-NHEJ (42). AltEJ is largely inactive in normal cells, but in quickly dividing cancer cells, altEJ may be increased to handle the increased level of DNA damage and, as a result, generate more mutations as by-products. Although the cell cycle dependency of altEJ is not clear, it is possible that HRR-deficient cells use altEJ mainly in S or G2 phases, while c-NHEJ defects may increase altEJ in G1 phase. In addition, host cell type, chromosomal location, and epigenetic modification are also important factors for pathway competence at DSBs, which has been reviewed elsewhere (43).

## Exploiting DNA Repair Deficits in Cancer Cells

Many DNA repair genes are tumor suppressors, and are frequently mutated during tumor progression. Loss of functions in some DNA repair genes increases compensating mechanisms of repair that may expose a targetable “Achilles heel.” Synthetic lethality, which is defined as a cytotoxic response to the loss or inhibition of a gene or pathway that only happens in the presence of another specific genetic deficit, can specifically target cancer cells containing a defect in a DNA repair gene. The clinical application of synthetic lethality is best exemplified using PARP inhibitors in cells that have germ line or somatic mutations in *BRCA1* or *BRCA2* (44, 45). PARP inhibition results in the accumulation of single-strand breaks (SSB) that produce DSBs upon collision with a DNA replication fork, which require HRR to repair. This is one mechanism by which BRCA1/2-deficient cancers are highly sensitized to PARP inhibition, although other functions of PARP may contribute as well. For example, PARP is directly involved in detoxifying endogenous ROS (46, 47), while BRCA1 down-regulates cellular levels of ROS (48). Thus, BRCA deficient cells may be more sensitive to increased cytotoxic ROS that result from PARP inhibition. Although the clinical benefits of PARP inhibition are clear in BRCA1/2-deficient cancers, tumors with HRR defects in the absence of BRCA gene mutations (i.e., BRCAness) are also responsive to PARP inhibition (49). Hence, mutation of genes that are directly or indirectly involved in HRR or the Fanconi anemia pathway, a DNA repair pathway that intersects HRR, can contribute to so-called BRCAness, i.e., tumors that behave as if *BRCA1* or *BRCA2* are mutated (49). Biomarkers to assess BRCAness, which include transcriptional signatures, genomic scars, or levels of Rad51 foci, are under intensive investigation (50–52).

## Synthetic Lethality Between altEJ and Other DSB Repair Pathways

Besides its key role in SSB repair, PARP1 is also a critical component of altEJ; thus, PARP inhibition can cause synthetic lethality in tumors that rely on altEJ (25). Other altEJ components are promising therapeutic targets for tumors that depend on this repair pathway. Indeed Pol θ -mediated end joining becomes critical when either HRR or c-NHEJ fails. A recent genetic screen reported that both Pol θ and another altEJ component, the structure-specific endonuclease FEN1, are synthetic lethal with BRCA2 (53). The potential synergy of HRR and altEJ is indicated by embryonic lethality of combined loss of *Brca1/Fancd2* and *Polq* in mice (28, 41). Depletion of Pol θ in human cancer cells deficient in HRR due to absence of BRCA1/2 increases chromosomal aberrations and impaired cell survival (28). Pol θ loss also increases HRR-impaired cells sensitivity to DNA-damage (41).

Moreover, there is growing evidence that defects in HRR can lead to increased dependence on altEJ for repair, particularly in the context of fork stalling during replication stress (26, 54). In addition to its relationship with HRR, altEJ may also be synthetic lethal with c-NHEJ. Combined deletion of *POLQ* and Ku70, as well as *POLQ* and *53BP1*, leads to markedly reduced cell proliferation and survival associated with excessive end resection and chromosomal aberrations (55). Notably, *POLQ* is highly expressed in a subset of cancer types (56, 57) and its expression is associated with poor prognosis (58, 59).

## DNA Repair in Context: the Tumor Microenvironment

Cancer cells react to endogenous (e.g., replication stress) or exogenous (e.g., radiation, chemotherapy) DNA damage in the context of their TME. The TME plays an important role in determining cancer clinical behavior and progression, and can influence cancer cell response to therapy (60, 61). Components of the TME include cellular constituents of bone-marrow derived cells, fibroblasts and vessels, insoluble extracellular matrix, and soluble cytokines and chemokines (62). These TME components closely collaborate with cancer cells for development of a neoplastic phenotype. In this “team,” frequent interactions and crosstalk in a complicated signaling network unite them as a whole. Better understanding of “tumor as a whole” could provide information about the optimal use of therapies, and improve the development of personalized therapy based on integrated features of a tumor derived from both cancer cells and TME composition.

The influence of TME conditions on DNA repair is complex. Although DNA repair is largely regulated through autonomous signaling cascades, tissue-wide stress responses from DNA damage may be networked among tumor cells, stromal cells, and other TME components. TME composition influences DNA repair efficiency by transmitting inter- and intra-cellular signals in a tissue-specific fashion. Many TME factors, which include cytokines, extracellular matrix, stromal cells, hypoxia, and inflammation, are known to modulate DNA repair efficiency (63–68).

Here we focus on TGFβ, a highly pleiotropic cytokine and a canonical tumor suppressor. Because TGFβ suppresses epithelial cell cycle progression, all carcinomas must escape TGFβ growth regulation (69). Notably, high TGFβ expression and signaling is associated with poor prognosis in multiple cancer types (52, 70) because TGFβ becomes a tumor promoter that is involved in tumor progression by modifying the TME, suppressing immune response, and promoting metastasis (71). Due to these critical functions and association with poor outcomes, TGFβ is an intriguing therapeutic target in clinical trials (72, 73).

## TGFβ Biology

Understanding the biology of TGFβ is rooted in understanding when and where it is active. There are three mammalian TGFβ isoforms, each encode a polypeptide that is cleaved intracellularly to form a roughly 24 kD TGFβ that is non-covalently associated with an 80 kD dimer of its pre-pro peptide, called latency associated peptide (LAP). This complex, TGFβ and LAP, is secreted as the latent TGFβ complex and is often sequestered in forms bound to extracellular matrix. TGFβ canonical signaling is initiated upon the release of TGFβ ligand from its latent complex, and subsequent binding to the type II TGFβ receptor (TβRII), which causes recruitment and phosphorylation of type I receptor (TβRI). Activated TβRI kinase phosphorylates the carboxy-terminal serine residue of the mothers against DPP Homolog proteins SMAD2 or SMAD3, which induces oligomerization of SMAD2 or SMAD3 with SMAD4, and DNA binding of the complex to mediate transcriptional activation or repression of target genes. TGFβ also transduces signals through non-canonical signaling pathways, such as MAPK/ERK, PI3K/AKT, Rho/Rock. Activation of TGFβ is highly controlled in normal tissues. In cancers, TGFβ signaling is highly dysregulated (70, 74, 75). Defective TGFβ signaling, which can be caused by mutations in *SMAD4* and *TGFBR2*, are frequent in certain types of cancer (52, 73).

Both cancer cells and stromal cells produce TGFβ that may, when activated, elicit paracrine or autocrine signaling to stimulate fibroblasts, endothelial cells and immune cells that further alter the TME. Moreover, TGFβ is a potent immunosuppressive cytokine involved in shaping TME by inhibiting the activation and function of T cells (76, 77), inducing immune suppressive myeloid cells (78), as well as by other multifaceted mechanisms (79–81). These complex interactions are just a few of TGFβ's roles in the TME.

An unexpected role for TGFβ, an intrinsically extracellular signal, is response to intracellular DNA damage. Glick et al. (82) was likely the first study to implicate TGFβ in the cellular response to DNA damage. They showed that *Tgfb1* null murine keratinocytes were highly genomically unstable, independent of G1 arrest and p53 function (82). Consistent with a role of TGFβ in maintaining genomic stability, Maxwell et al. (83) demonstrated more centrosome aberrations and aneuploidy in irradiated *Tgfb1*-null compared to TGFβ-competent keratinocytes. Notably, this effect is phenocopied by TGFβ inhibition in human epithelial cells (83). In a subsequent study, Glick and colleagues revealed a DNA repair deficit due to hypermethylated O(6)-methylguanine DNA methyltransferase (MGMT) that affected the DDR of *Tgfb1*-null keratinocytes (84). Similarly, Kirshner et al. (85) showed that inhibiting TGFβ signaling attenuates DDR by compromising the function of ATM, the DDR kinase involved in DSB recognition (85). Consequently, pharmaceutical inhibition of TβRI kinase or knockout of *Tgfb1* reduces ATM autophosphorylation and phosphorylation of its substrates, e.g., p53, Chk2, and Rad17, inhibits formation of radiation-induced γH2AX foci, and increases radiosensitivity (85). In support of this, Wiegman et al. (86) showed that exogenous TGFβ stimulates ATM and p53 phosphorylation in irradiated cells in a SMAD-independent fashion (86). Notably, TGFβ inhibition also reduces *LIG4* expression, which is required in c-NHEJ (87).

Nucleotide excision repair (NER) is a versatile DNA repair pathway that eliminates a wide variety of helix-distorting base lesions induced by environmental carcinogenic sources. UVB radiation downregulates E-cadherin, a cell adhesion protein, in mouse skin and skin tumors whereas inhibiting the TGFβ pathway in these cells increases the NER of UV-induced DNA damage (88). E-cadherin inhibition in keratinocytes suppresses NER through activating the TGF-β pathway and increasing *TGF*β*1* mRNA levels. Interestingly, TGFβ is activated by ionizing radiation and in turn, promotes epithelial-mesenchymal transition characterized by loss of E-cadherin (89). Treatment of cells with exogenous TGFβ enhances NER of DNA damage formed by polycyclic aromatic hydrocarbons and UVC radiation independent of the cell cycle (90).

Consistent with role of TGFβ in DDR, the SMAD proteins, which are the critical transducers of TGFβ intracellular signaling, are involved in DSB repair. Both pSmad2 and Smad7 can co-localize with nuclear γH2AX foci at DSB, while pSmad2 foci formation is ATM dependent (91). Studies in *Smad4* conditional knockout mice confirmed that TGFβ is critical in maintaining genomic stability through regulation of genes in the Fanconi anemia/BRCA DNA repair pathway (92). SMAD4 suppresses a micro RNA, miR182, which inhibits FOXO3, which is required for ATM kinase activity (32, 52). MiR182 also suppresses BRCA1 expression (93, 94). Hence, TGFβ signaling through SMAD4 promotes HRR in part by suppressing miR182 (52). C-NHEJ is also partially compromised because LIG4 expression and ATM activity are reduced once TGFβ signaling is blocked (85, 87).

Although tumors must evade TGFβ growth control, at the time of clinical appearance, many tumors maintain signaling competency. Indeed, squamous cell carcinomas may take advantage of TGFβ signaling to maintain a sub-population of cells at a quiescent state for chemo-resistance (95). Moreover, the high TGFβ activity of TME could promote tumor-intrinsic resistance to cytotoxic agents due to its role in DNA damage recognition and repair. If so, pharmacological TGFβ blockade could sensitize certain tumors to radiation and other cytotoxic therapies. Exploration of brain, breast, and lung cancer pre-clinical models is consistent with this concept, since TGFβ inhibition radiosensitized 38 of 43 murine and human cancer cell lines *in vitro* (85, 96–98). Because TGFβ provides extrinsic control of several aspects of intracellular DNA repair pathway competency, one prediction is that tumors that are insensitive to TGFβ can be exploited by targeting their deficiency in DNA repair.

## TGFβ Signaling Regulates DNA Repair Pathway Competency

The contribution of TGFβ signaling as a barrier compromising therapeutic response to cytotoxic therapy is exemplified by HPV-positive (HPV+) HNSCC (52), which have much better (70%) survival at 5 years compared to HPV-negative (HPV-) cancers that attain only 30% survival, even when HNSCC location and stage are similar. The considerable difference in outcomes has stimulated significant interest based on the idea that the mechanism of sensitivity of HPV+ cancer to standard-of-care chemoradiation therapy, which could provide insights that can be therapeutically exploited to achieve better response in HPV- cancer.

Consistent with a cell-intrinsic effect, HPV+ cancer cell lines exhibit altered expression of DNA repair proteins (99) and increased sensitivity to cytotoxic therapy (100, 101). Most research has focused on oncogenic impairment of p53 and retinoblastoma (Rb) proteins by HPV E6 or E7, respectively. However, HPV proteins E5, E6, and E7 target both the type I and II TGFβ receptors and SMAD 2, 3, and 4, the transducers of ligand binding, for degradation (102, 103). We examined the impact of HPV on TGFβ signaling in HNSCC at multiple levels. Functionally, TGFβ induced phosphorylation of SMAD2 (pSMAD), indicative of signaling competency, is significantly reduced in HPV+ cell line, patient derived xenografts, and primary tumor explants, compared to HPV− specimens (52). Notably, HPV+ specimens in a HNSCC tumor array with 130 HPV− and 65 HPV+ samples exhibit less pSMAD compared to HPV− specimens. In addition, TCGA HPV+ tumors are identified by low activity (243 HPV− vs. 36 HPV+ ones) of a TGFβ pathway signature, which contains 50 TGFβ-regulated genes that were derived from epithelial cells chronically stimulated or inhibited for the corresponding pathway.

Interestingly, Wang and colleagues engineered a conditional *Smad4* deletion in oral mucosa that gave rise to spontaneous HNSCC accompanied by high rates of genomic instability (92). Subsequent studies by this group showed that *Smad4* deletion leads to decreased Brca1 expression in mice and that loss of SMAD4 protein correlates with decreased BRCA1 and RAD51 proteins in human HNSCC. BRCA1 is crucial for HRR during S-phase/G2, acts upon the cell cycle machinery, and affects gene expression and cell fate decisions via chromatin remodeling and transcriptional activity (104). Wang and colleagues showed that BRCA1 is transcriptionally down-regulated by SMAD4-dependent CtPB1 (105). A second, more direct route by which TGFβ controls of BRCA1 is via TGFβ suppression of miR-182, which targets BRCA1 message stability and translation in mouse and human cells. Thus, pharmacologically or genetically compromised TGFβ increase levels of miR-182 and consequently suppress BRCA1 (94).

Consistent with our earlier studies in breast, lung and brain cancer cells (85, 96–98), HPV+ HNSCC cell lines were more sensitive to radiation than HPV− cell lines (52). Indeed, the degree of pSMAD response to TGFβ and cellular radiosensitivity are highly correlated. Radiation sensitivity reflects the cumulative damage and inherent capacity to repair the damage based on the cells ability to recognize DNA damage, assemble the repair machinery, and execute repair; abrogation of any of these components decreases cell survival. As noted above, BRCA1 is critical for HRR-mediated DNA repair. HRR requires RAD51 binding to 3′-single-stranded DNA overhangs from processed DSB and strand pairing; thus, the formation of RAD51 foci is evidence of HRR. Significantly, fewer RAD51 foci are formed in HPV- HNSCC cells and tumor specimens if TGFβ is pharmacologically blocked, which is not observed in HPV+ HNSCC cells.

A deficiency in HRR should increase the proportion of cells that are killed in response to PARPi. As predicted, TGFβ-unresponsive HPV+ cell lines are more sensitive to olaparib alone compared to TGFβ-responsive HPV- cancer cells, which were sensitized 4-fold by TGFβ inhibition (52).

Loss of effective HRR can activate altEJ, which competes with HRR for repair of DSBs in S-phase (41) and/or acts as backup repair when HRR or c-NHEJ are compromised (26). As mentioned earlier, altEJ requires PARP1 and Pol θ, the product of the *POLQ* gene (28). To evaluate altEJ, we established TGFβ-responsive cells with a reporter construct detecting altEJ events (106). As expected, a specific PARP1 inhibitor reduced altEJ events, while TGFβ inhibitors significantly increased altEJ events.

**Figure 2:**
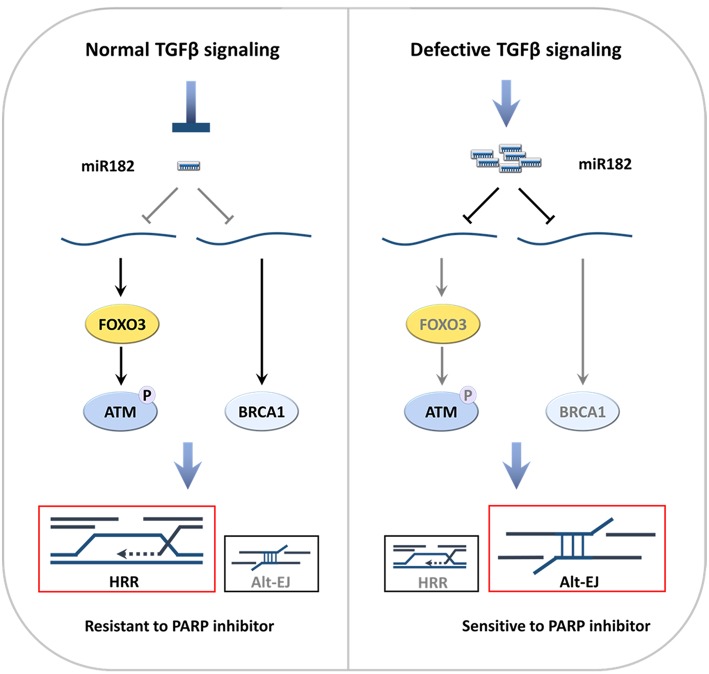
Defective TGFβ signaling in HNSCC increases altEJ (52). **(A)** TGFβ signaling suppresses miR182, which suppresses expression of *BRCA1* and *FOXO3*. DNA damage elicits activates ATM autophosphorylation and its phosphorylation of BRCA1, which gives rise to use of HRR in S-phase. **(B)** In contrast, deficient TGFβ signaling, which can be caused by HPV infection, TGFβ receptor kinase inhibitors (TBRi) or *SMAD4* mutations (*SMAD4* mut), leads to increased miR182 that suppresses *BRCA1* and *FOXO3*. Loss of FOXO3 inhibits ATM auto-activation, which together with decreased BRCA1, impedes HRR. This is accompanied by increased altEJ.

Thus, either pharmaceutical blockade of TGFβ signaling in HPV− cells or intrinsic-defects in TGFβ signaling in HPV+ cells shifts DSB repair to altEJ (Figure 2). This shift may result from decreased implementation of HRR and c-NHEJ, or may indicate an increase in altEJ competency. Notably, *BRCA1* heterozygous cells exhibit preferential use of altEJ (107), suggesting that the decrease in BRCA1 that occurs upon loss of TGFβ signaling phenocopies the genetic *BRCA1* loss. As c-NHEJ is also partially compromised because LIG4 expression and ATM activity are reduced (85, 87), cells with deficient TGFβ signaling may increase altEJ to compensate for a deficiency in both HRR and c-NHEJ.

TGFβ-unresponsive cells that depend on altEJ are still capable of rejoining most DSBs, which hinders the maximal cytotoxic response to DNA damage. As mentioned above, recent studies demonstrate that Pol θ is required in altEJ (28, 41). HRR-deficient ovarian and breast cancers exhibit increased *POLQ* expression, perhaps indicative of altEJ (41). Consistent with this, *POLQ* expression is increased in HPV+ vs. HPV− HNSCC TCGA (52). Notably, *POLQ* shRNA expressing HPV− cells treated with TGFβ inhibitors were more sensitive DNA damage, supporting the idea that Pol θ-mediated altEJ is increasingly used when TGFβ signaling is abrogated. These data suggest that combining altEJ inhibitors with pharmaceutical blockade of TGFβ signaling would induce synthetic lethality, thus creating a novel route that could boost the sensitivity of TGFβ-active tumors to therapies that involve DNA damage. Moreover, since altEJ is a backup mechanism that operates preferentially in cancer cells, inhibiting altEJ may pre-dominantly sensitize tumor cells and spare normal cells. Alternatively, one might select cancers that are defective in TGFβ to target drugs that interfere with altEJ, since these drugs will both improve the treatment effectiveness and the toxicity profile.

The importance of alt-EJ in TGFβ-deficient cells discovered in HNSCC raises several questions. We are following this lead to determine if the same mechanism is evident in other cancers in which TGFβ signaling is compromised. Almost all cervical cancers are HPV positive, which we would expect to show similar DDR choices as HNSCC. However, TGFβ signaling is compromised by various mechanisms in many cancers (108). Our analysis across the spectrum of cancers suggest that there is probably a generic mechanism (unpublished). We also find that the repair shift to altEJ in TGFβ-deficient cells occurs independent of miR-182. Further studies are necessary to decipher the underlying mechanisms.

Although most evidence supports TGFβ as a factor enforcing DNA-repair proficiency to protect against genomic instability, conflicting evidence about the role of TGFβ in DDR also exists (109–112). For example, Pal et al. reported that TGFβ hinders DSB repair in cancer stem cells by reducing HRR gene expression, which was proposed to heighten genetic diversity and adaptability of cancer stem cells (112). This subpopulation may regulate DDR differently, but it is interesting that in another study, glioblastoma cancer stem cells make 5-fold more TGFβ than bulk cultures (97). Considering that TGFβ is a pleotropic cytokine, the extensive technical and conditional differences in these studies may lead to a different conclusion.

For example, TGFβ signaling induces squamous cell carcinoma cancer stem cells quiescence, which would be expected to affect repair pathway competency in a manner that contributes to chemo-resistance (95). However, many cancer cells have escaped TGFβ cell growth control, as stated above. For example, in our HNSCC study, the cell cycle was not significantly changed upon TGFβ stimulation or inhibition of HNSCC cell lines (52), which indicates that TGFβ can regulate DNA repair pathway by mechanisms independent of cell cycle effects.

## Outlook

Intensive investigation of the TME has advanced our knowledge about the tumor as a whole, whereas in-depth analysis of DDR now provides mechanisms of DNA repair strategies and their implementation in cancer cells. How DDR is executed in different tumors is an area of growing complexity. The highly heterogeneous TME is a function of cancer cell genetics, epigenetics, host cell composition that result in complicated signaling networks. However, opportunities also lie in these challenges. DDR-related signaling pathways regulated by the TME components may contain biomarkers for cancer stratification, as well as molecular or cellular targets for drug development, which deserves significant investigation.

## Author Contributions

QL, JM, and MB-H wrote the review. TH and KL reviewed and revised the manuscript.

### Conflict of Interest Statement

The authors declare that the research was conducted in the absence of any commercial or financial relationships that could be construed as a potential conflict of interest.

## References

[1] Lindahl T Barnes DE. Repair of endogenous DNA damage. Cold Spring Harb Symp Quant Biol. (2000) 65:127–33. 10.1101/sqb.2000.65.127 12760027

[2] Tubbs A Nussenzweig A. Endogenous DNA damage as a source of genomic instability in cancer. Cell. (2017) 168:644–56. 10.1016/j.cell.2017.01.002 28187286 PMC6591730

[3] Krejci L Altmannova V Spirek M Zhao X. Homologous recombination and its regulation. Nucleic Acids Res. (2012) 40:5795–818. 10.1093/nar/gks270 22467216 PMC3401455

[4] Beucher A Birraux J Tchouandong L Barton O Shibata A Conrad S. ATM and Artemis promote homologous recombination of radiation-induced DNA double-strand breaks in G2. EMBO J. (2009) 28:3413–27. 10.1038/emboj.2009.276 19779458 PMC2752027

[5] Takata M Sasaki MS Sonoda E Morrison C Hashimoto M Utsumi H. Homologous recombination and non-homologous end-joining pathways of DNA double-strand break repair have overlapping roles in the maintenance of chromosomal integrity in vertebrate cells. EMBO J. (1998) 17:5497–508. 10.1093/emboj/17.18.5497 9736627 PMC1170875

[6] Bozzella M Seluanov A Gorbunova V DNA repair by nonhomologous end joining and homologous recombination during cell cycle in human cells AU - Mao, Zhiyong. Cell Cycle. (2008) 7:2902–6. 10.4161/cc.7.18.6679 18769152 PMC2754209

[7] Meek K Dang V Lees-Miller SP. DNA-PK: the means to justify the ends? Adv Immunol. (2008) 99:33–58. 10.1016/S0065-2776(08)00602-0 19117531

[8] Stiff T Walker SA Cerosaletti K Goodarzi AA Petermann E Concannon P ATR-dependent phosphorylation and activation of ATM in response to UV treatment or replication fork stalling. Embo J. (2006) 25:5775–82. 10.1038/sj.emboj.7601446 17124492 PMC1698893

[9] Bebenek K Pedersen LC Kunkel TA. Structure-function studies of DNA polymerase lambda. Biochemistry. (2014) 53:2781–92. 10.1021/bi4017236 24716527 PMC4018081

[10] Moon AF Pryor JM Ramsden DA Kunkel TA Bebenek K Pedersen LC. Sustained active site rigidity during synthesis by human DNA polymerase mu. Nat Struct Mol Biol. (2014) 21:253–60. 10.1038/nsmb.2766 24487959 PMC4164209

[11] Pryor JM Conlin MP Carvajal-Garcia J Luedeman ME Luthman AJ Small GW. Ribonucleotide incorporation enables repair of chromosome breaks by nonhomologous end joining. Science. (2018) 361:1126–9. 10.1126/science.aat2477 30213916 PMC6252249

[12] Ahnesorg P Smith P Jackson SP. XLF interacts with the XRCC4-DNA ligase IV complex to promote DNA nonhomologous end-joining. Cell. (2006) 124:301–13. 10.1016/j.cell.2005.12.031 16439205

[13] Betermier M Bertrand P Lopez BS. Is non-homologous end-joining really an inherently error-prone process? PLoS Genet. (2014) 10:e1004086. 10.1371/journal.pgen.1004086 24453986 PMC3894167

[14] Sishc BJ Davis AJ. The role of the core non-homologous end joining factors in carcinogenesis and cancer. Cancers. (2017) 9:E81. 10.3390/cancers9070081 28684677 PMC5532617

[15] Powell SN Kachnic LA. Roles of BRCA1 and BRCA2 in homologous recombination, DNA replication fidelity and the cellular response to ionizing radiation. Oncogene. (2003) 22:5784–91. 10.1038/sj.onc.1206678 12947386

[16] Li X Heyer WD. Homologous recombination in DNA repair and DNA damage tolerance. Cell Res. (2008) 18:99–113. 10.1038/cr.2008.1 18166982 PMC3087377

[17] Sartori AA Lukas C Coates J Mistrik M Fu S Bartek J. Human CtIP promotes DNA end resection. Nature. (2007) 450:509–14. 10.1038/nature06337 17965729 PMC2409435

[18] Zhu Z Chung WH Shim EY Lee SE Ira G. Sgs1 helicase and two nucleases Dna2 and Exo1 resect DNA double-strand break ends. Cell. (2008) 134:981–94. 10.1016/j.cell.2008.08.037 18805091 PMC2662516

[19] Nimonkar AV Genschel J Kinoshita E Polaczek P Campbell JL Wyman C. BLM-DNA2-RPA-MRN and EXO1-BLM-RPA-MRN constitute two DNA end resection machineries for human DNA break repair. Genes Dev. (2011) 25:350–62. 10.1101/gad.2003811 21325134 PMC3042158

[20] Chen H Lisby M Symington LS. RPA coordinates DNA end resection and prevents formation of DNA hairpins. Mol Cell. (2013) 50:589–600. 10.1016/j.molcel.2013.04.032 23706822 PMC3855855

[21] Langberg CW Hauer-Jensen M Sung C-C Kane CJM. Expression of fibrogenic cytokines in rat small intestine after fractionated irradiation. Radiother Oncol. (1994) 32:29–36. 10.1016/0167-8140(94)90446-4 7938676

[22] Yang H Jeffrey PD Miller J Kinnucan E Sun Y Thoma NH. BRCA2 function in DNA binding and recombination from a BRCA2-DSS1-ssDNA structure. Science. (2002) 297:1837–48. 10.1126/science.297.5588.1837 12228710

[23] Lord CJ Ashworth A. PARP inhibitors: synthetic lethality in the clinic. Science. (2017) 355:1152–8. 10.1126/science.aam7344 28302823 PMC6175050

[24] Jasin M Rothstein R. Repair of strand breaks by homologous recombination. Cold Spring Harb Perspect Biol. (2013) 5:a012740. 10.1101/cshperspect.a012740 24097900 PMC3809576

[25] Frit P Barboule N Yuan Y Gomez D Calsou P. Alternative end-joining pathway(s): bricolage at DNA breaks. DNA Repair. (2014) 17:81–97. 10.1016/j.dnarep.2014.02.007 24613763

[26] Iliakis G Murmann T Soni A. Alternative end-joining repair pathways are the ultimate backup for abrogated classical non-homologous end-joining and homologous recombination repair: Implications for the formation of chromosome translocations. Mutat Res Genet Toxicol Environ Mutagen. (2015) 793:166–75. 10.1016/j.mrgentox.2015.07.001 26520387

[27] Robert I Dantzer F Reina-San-Martin B. Parp1 facilitates alternative NHEJ, whereas Parp2 suppresses IgH/c-myc translocations during immunoglobulin class switch recombination. J Exp Med. (2009) 206:1047–56. 10.1084/jem.20082468 19364882 PMC2715026

[28] Mateos-Gomez PA Gong F Nair N Miller KM Lazzerini-Denchi E Sfeir A. Mammalian polymerase theta promotes alternative NHEJ and suppresses recombination. Nature. (2015) 518:254–7. 10.1038/nature14157 25642960 PMC4718306

[29] Li X Lu Y Liang K Pan T Mendelsohn J Fan Z. Requirement of hypoxia-inducible factor-1alpha down-regulation in mediating the antitumor activity of the anti-epidermal growth factor receptor monoclonal antibody cetuximab. Mol Cancer Ther. (2008) 7:1207–17. 10.1158/1535-7163.MCT-07-2187 18483308

[30] Ferguson DO Sekiguchi JM Chang S Frank KM Gao Y Depinho RA. The nonhomologous end-joining pathway of DNA repair is required for genomic stability and the suppression of translocations. Proc Natl Acad Sci USA. (2000) 97:6630–3. 10.1073/pnas.110152897 10823907 PMC18682

[31] Simsek D Jasin M. Alternative end-joining is suppressed by the canonical NHEJ component Xrcc4-ligase IV during chromosomal translocation formation. Nat Struct Mol Biol. (2010) 17:410–6. 10.1038/nsmb.1773 20208544 PMC3893185

[32] Tsai W-B Chung YM Takahashi Y Xu Z Hu MCT. Functional interaction between FOXO3a and ATM regulates DNA damage response. Nat Cell Biol. (2008) 10:460–7. 10.1038/ncb1709 18344987 PMC2674111

[33] Yu X Chen J. DNA damage-induced cell cycle checkpoint control requires CtIP, a phosphorylation-dependent binding partner of BRCA1 C-terminal domains. Mol Cell Biol. (2004) 24:9478–86. 10.1128/MCB.24.21.9478-9486.2004 15485915 PMC522253

[34] Chen L Nievera CJ Lee AY Wu X. Cell cycle-dependent complex formation of BRCA1.CtIP.MRN is important for DNA double-strand break repair. J Biol Chem. (2008) 283:7713–20. 10.1074/jbc.M710245200 18171670

[35] Isono M Niimi A Oike T Hagiwara Y Sato H Sekine R. BRCA1 directs the repair pathway to homologous recombination by promoting 53BP1 dephosphorylation. Cell Rep. (2017) 18:520–32. 10.1016/j.celrep.2016.12.042 28076794

[36] Noordermeer SM Adam S Setiaputra D Barazas M Pettitt SJ Ling AK. The shieldin complex mediates 53BP1-dependent DNA repair. Nature. (2018) 560:117–21. 10.1038/s41586-018-0340-7 30022168 PMC6141009

[37] Gupta R Somyajit K Narita T Maskey E Stanlie A Kremer M. DNA Repair network analysis reveals shieldin as a key regulator of NHEJ and PARP inhibitor sensitivity. Cell. (2018) 173:972–988.e923. 10.1016/j.cell.2018.03.050 29656893 PMC8108093

[38] Paddock MN Bauman AT Higdon R Kolker E Takeda S Scharenberg AM. Competition between PARP-1 and Ku70 control the decision between high-fidelity and mutagenic DNA repair. DNA Repair. (2011) 10:338–43. 10.1016/j.dnarep.2010.12.005 21256093 PMC4079052

[39] Ahrabi S Sarkar S Pfister SX Pirovano G Higgins GS Porter AC. A role for human homologous recombination factors in suppressing microhomology-mediated end joining. Nucleic Acids Res. (2016) 44:5743–57. 10.1093/nar/gkw326 27131361 PMC4937322

[40] Dynan WS Yoo S. Interaction of Ku protein and DNA-dependent protein kinase catalytic subunit with nucleic acids. Nucleic Acids Res. (1998) 26:1551–9. 10.1093/nar/26.7.1551 9512523 PMC147477

[41] Ceccaldi R Liu JC Amunugama R Hajdu I Primack B Petalcorin MI. Homologous-recombination-deficient tumours are dependent on Poltheta-mediated repair. Nature. (2015) 518:258–62. 10.1038/nature14184 25642963 PMC4415602

[42] Arnoult N Correia A Ma J Merlo A Garcia-Gomez S Maric M. Regulation of DNA repair pathway choice in S and G2 phases by the NHEJ inhibitor CYREN. Nature. (2017) 549:548–52. 10.1038/nature24023 28959974 PMC5624508

[43] Kakarougkas A Jeggo PA. DNA DSB repair pathway choice: an orchestrated handover mechanism. Br J Radiol. (2014) 87:20130685. 10.1259/bjr.20130685 24363387 PMC4064598

[44] Bryant HE Schultz N Thomas HD Parker KM Flower D Lopez E. Specific killing of BRCA2-deficient tumours with inhibitors of poly(ADP-ribose) polymerase. Nature. (2005) 434:913–7. 10.1038/nature03443 15829966

[45] Farmer H Mccabe N Lord CJ Tutt AN Johnson DA Richardson TB. Targeting the DNA repair defect in BRCA mutant cells as a therapeutic strategy. Nature. (2005) 434:917–21. 10.1038/nature03445 15829967

[46] Liu Q Gheorghiu L Drumm M Clayman R Eidelman A Wszolek MF. PARP-1 inhibition with or without ionizing radiation confers reactive oxygen species-mediated cytotoxicity preferentially to cancer cells with mutant TP53. Oncogene. (2018) 37:2793–805. 10.1038/s41388-018-0130-6 29511347 PMC5970015

[47] Marcar L Bardhan K Gheorghiu L Dinkelborg P Pfaffle H Liu Q. Acquired resistance of EGFR-mutated lung cancer to tyrosine kinase inhibitor treatment promotes PARP inhibitor sensitivity. Cell Rep. (2019) 27:3422–32.e3424. 10.1016/j.celrep.2019.05.058 31216465 PMC6624074

[48] Saha T Rih JK Rosen EM. BRCA1 down-regulates cellular levels of reactive oxygen species. FEBS Lett. (2009) 583:1535–43. 10.1016/j.febslet.2009.04.005 19364506 PMC2744635

[49] Lord CJ Ashworth A. BRCAness revisited. Nat Rev Cancer. (2016) 16:110–20. 10.1038/nrc.2015.21 26775620

[50] Willers H Gheorghiu L Liu Q Efstathiou JA Wirth LJ Krause M. DNA damage response assessments in human tumor samples provide functional biomarkers of radiosensitivity. Semin Radiat Oncol. (2015) 25:237–50. 10.1016/j.semradonc.2015.05.007 26384272 PMC4575410

[51] Ganguly B Dolfi SC Rodriguez-Rodriguez L Ganesan S Hirshfield KM. Role of biomarkers in the development of PARP inhibitors. Biomark Cancer. (2016) 8:15–25. 10.4137/BIC.S36679 26997874 PMC4786099

[52] Liu Q Ma L Jones T Palomero L Pujana MA Martinez-Ruiz H Subjugation of TGFb signaling by human papilloma virus in head and neck squamous cell carcinoma shifts DNA repair from homologous recombination to alternative end joining. Clin Cancer Res. (2018) 24:6001–14. 10.1158/1078-0432.CCR-18-1346 30087144

[53] Mengwasser KE Adeyemi RO Leng Y Choi MY Clairmont C D'andrea AD. Genetic screens reveal FEN1 and APEX2 as BRCA2 synthetic lethal targets. Mol Cell. (2019) 73:885–99.e6. 10.1016/j.molcel.2018.12.008 30686591 PMC6892393

[54] Garcia-Closas M Couch FJ Lindstrom S Michailidou K Schmidt MK Brook MN. (2013). Genome-wide association studies identify four ER negative-specific breast cancer risk loci. Nat Genet. 45:392–8. 10.1038/ng.2561 23535733 PMC3771695

[55] Wyatt DW Feng W Conlin MP Yousefzadeh MJ Roberts SA Mieczkowski P. Essential roles for polymerase theta-mediated end joining in the repair of chromosome breaks. Mol Cell. (2016) 63:662–73. 10.1016/j.molcel.2016.06.020 27453047 PMC4992412

[56] Kawamura K Bahar R Seimiya M Chiyo M Wada A Okada S. DNA polymerase theta is preferentially expressed in lymphoid tissues and upregulated in human cancers. Int J Cancer. (2004) 109:9–16. 10.1002/ijc.11666 14735462

[57] Lemee F Bergoglio V Fernandez-Vidal A Machado-Silva A Pillaire MJ Bieth A. DNA polymerase theta up-regulation is associated with poor survival in breast cancer, perturbs DNA replication, and promotes genetic instability. Proc Natl Acad Sci USA. (2010) 107:13390–5. 10.1073/pnas.0910759107 20624954 PMC2922118

[58] Higgins GS Harris AL Prevo R Helleday T Mckenna WG Buffa FM. Overexpression of POLQ confers a poor prognosis in early breast cancer patients. Oncotarget. (2010) 1:175–84. 10.18632/oncotarget.124 20700469 PMC2917771

[59] Allera-Moreau C Rouquette I Lepage B Oumouhou N Walschaerts M Leconte E. DNA replication stress response involving PLK1, CDC6, POLQ, RAD51 and CLASPIN upregulation prognoses the outcome of early/mid-stage non-small cell lung cancer patients. Oncogenesis. (2012) 1:e30. 10.1038/oncsis.2012.29 23552402 PMC3503291

[60] Chan N Koch CJ Bristow RG. Tumor hypoxia as a modifier of DNA strand break and cross-link repair. Curr Mol Med. (2009) 9:401–10. 10.2174/156652409788167050 19519397

[61] Sun Y Campisi J Higano C Beer TM Porter P Coleman I. Treatment-induced damage to the tumor microenvironment promotes prostate cancer therapy resistance through WNT16B. Nat Med. (2012) 18:1359–68. 10.1038/nm.2890 22863786 PMC3677971

[62] Hanahan D Coussens LM. Accessories to the crime: functions of cells recruited to the tumor microenvironment. Cancer Cell. (2012) 21:309–22. 10.1016/j.ccr.2012.02.022 22439926

[63] Kumareswaran R Ludkovski O Meng A Sykes J Pintilie M Bristow RG. Chronic hypoxia compromises repair of DNA double-strand breaks to drive genetic instability. J Cell Sci. (2012) 125:189–99. 10.1242/jcs.092262 22266907

[64] Kidane D Chae WJ Czochor J Eckert KA Glazer PM Bothwell AL. Interplay between DNA repair and inflammation, and the link to cancer. Crit Rev Biochem Mol Biol. (2014) 49:116–39. 10.3109/10409238.2013.875514 24410153 PMC4300235

[65] Geiger-Maor A Guedj A Even-Ram S Smith Y Galun E Rachmilewitz J. Macrophages regulate the systemic response to DNA damage by a cell nonautonomous mechanism. Cancer Res. (2015) 75:2663–73. 10.1158/0008-5472.CAN-14-3635 25977329

[66] Scanlon SE Glazer PM. Multifaceted control of DNA repair pathways by the hypoxic tumor microenvironment. DNA Repair. (2015) 32:180–9. 10.1016/j.dnarep.2015.04.030 25956861 PMC4522377

[67] Centurione L Aiello FB. DNA repair and cytokines: TGF-beta, IL-6, and thrombopoietin as different biomarkers of radioresistance. Front Oncol. (2016) 6:175. 10.3389/fonc.2016.00175 27500125 PMC4956642

[68] Dickreuter E Eke I Krause M Borgmann K Van Vugt MA Cordes N. Targeting of beta1 integrins impairs DNA repair for radiosensitization of head and neck cancer cells. Oncogene. (2016) 35:1353–62. 10.1038/onc.2015.212 26073085

[69] Derynck R Akhurst RJ Balmain A TGF-b signaling in tumor suppression and cancer progression. Nature Genet. (2001) 29:117–29. 10.1038/ng1001-117 11586292

[70] Costanza B Umelo IA Bellier J Castronovo V Turtoi A Stromal modulators of TGFβ in cancer. J Clin Med. (2017) 6:7 10.3390/jcm6010007 PMC5294960 28067804

[71] Akhurst RJ TGFβ antagonists: why suppress a tumor suppressor? J Clin Invest. (2002) 109:1533–6. 10.1172/JCI0215970 12070299 PMC151022

[72] Akhurst RJ Hata A. Targeting the TGFbeta signalling pathway in disease. Nat Rev Drug Discov. (2012) 11:790–11. 10.1038/nrd3810 23000686 PMC3520610

[73] Neuzillet C Tijeras-Raballand A Cohen R Cros J Faivre S Raymond E. Targeting the TGFbeta pathway for cancer therapy. Pharmacol Ther. (2015) 147:22–31. 10.1016/j.pharmthera.2014.11.001 25444759

[74] Barcellos-Hoff MH Latency and activation in the regulation of TGFβ. J Mammary Gland Biol Neoplasia. (1996) 3:353–63. 10.1007/BF02017391 10887509

[75] Jobling MF Mott JD Finnegan MT Jurukovski V Erickson AC Walian PJ Isoform-specific activation of latent transforming growth factor beta (LTGFβ) by reactive oxygen species. Radiat Res. (2006) 166:839–48. 10.1667/RR0695.1 17149983

[76] Ghiringhelli F Menard C Terme M Flament C Taieb J Chaput N. CD4^+^CD25^+^ regulatory T cells inhibit natural killer cell functions in a transforming growth factor-beta-dependent manner. J Exp Med. (2005) 202:1075–85. 10.1084/jem.20051511 16230475 PMC2213209

[77] Da Cunha AP Wu HY Rezende RM Vandeventer T Weiner HL. *In vivo* anti-LAP mAb enhances IL-17/IFN-γ responses and abrogates anti-CD3-induced oral tolerance. Int Immunol. (2015) 27:73–82. 10.1093/intimm/dxu083 25194146 PMC4303004

[78] Gonzalez-Junca A Driscoll KE Pellicciotta I Du S Lo CH Roy R. Autocrine TGFβ is a survival factor for monocytes and drives immunosuppressive lineage commitment. Cancer Immunol Res. (2019) 7:306–20. 10.1158/2326-6066.CIR-18-0310 30538091 PMC6828175

[79] Li Z Zhang LJ Zhang HR Tian GF Tian J Mao XL. Tumor-derived transforming growth factor-β is critical for tumor progression and evasion from immune surveillance. Asian Pac J Cancer Prev. (2014) 15:5181–6. 10.7314/apjcp.2014.15.13.5181 25040972

[80] Yang L Moses HL. Transforming growth factor-β: tumor suppressor or promoter? are host immune cells the answer? Cancer Res. (2008) 68:9107–11. 10.1158/0008-5472.CAN-08-2556 19010878 PMC2741321

[81] Wrzesinski SH Wan YY Flavell RA. Transforming growth factor-beta and the immune response: implications for anticancer therapy. Clin Cancer Res. (2007) 13:5262–70. 10.1158/1078-0432.CCR-07-1157 17875754

[82] Glick AB Weinberg WC Wu IH Quan W Yuspa SH. Transforming growth factor beta 1 suppresses genomic instability independent of a G1 arrest, p53, and Rb. Cancer Res. (1996) 56:3645–50. 8706000

[83] Maxwell CA Fleisch MC Costes SV Erickson AC Boissiere A Gupta R. Targeted and nontargeted effects of ionizing radiation that impact genomic instability. Cancer Res. (2008) 68:8304–11. 10.1158/0008-5472.CAN-08-1212 18922902

[84] Yamada H Vijayachandra K Penner C Glick A Increased sensitivity of transforming growth factor (TGF) β1 null cells to alkylating agents reveals a novel link between TGFbeta signaling and O(6)-methylguanine methyltransferase promoter hypermethylation. J Biol Chem. (2001) 276:19052–8. 10.1074/jbc.M100615200 11262404

[85] Kirshner J Jobling MF Pajares MJ Ravani SA Glick AB Lavin MJ. Inhibition of transforming growth factor-beta1 signaling attenuates ataxia telangiectasia mutated activity in response to genotoxic stress. Cancer Res. (2006) 66:10861–9. 10.1158/0008-5472.CAN-06-2565 17090522

[86] Wiegman EM Blaese MA Loeffler H Coppes RP Rodemann HP TGFbeta-1 dependent fast stimulation of ATM and p53 phosphorylation following exposure to ionizing radiation does not involve TGFβ-receptor I signalling. Radiother Oncol. (2007) 83:289–95. 10.1016/j.radonc.2007.05.013 17560675

[87] Kim MR Lee J An YS Jin YB Park IC Chung E. TGFβ1 protects cells from γ-IR by enhancing the activity of the NHEJ repair pathway. Mol Cancer Res. (2015) 13:319–29. 10.1158/1541-7786.MCR-14-0098-T 25319009

[88] Qiang L Shah P Barcellos-Hoff MH He YY. TGF-β signaling links E-cadherin loss to suppression of nucleotide excision repair. Oncogene. (2016) 35:3293–302. 10.1038/onc.2015.390 26477308 PMC4837109

[89] Andarawewa KL Erickson AC Chou WS Costes SV Gascard P Mott JD Ionizing radiation predisposes nonmalignant human mammary epithelial cells to undergo transforming growth factor b induced epithelial to mesenchymal transition. Cancer Res. (2007) 67:8662–70. 10.1158/0008-5472.CAN-07-1294 17875706

[90] Zheng H Jarvis IWH Bottai M Dreij K Stenius U. TGF beta promotes repair of bulky DNA damage through increased ERCC1/XPF and ERCC1/XPA interaction. Carcinogenesis. (2019) 40:580–91. 10.1093/carcin/bgy156 30418489

[91] Wang M Saha J Hada M Anderson JA Pluth JM O'neill P. Novel smad proteins localize to IR-induced double-strand breaks: interplay between TGFβ and ATM pathways. Nucleic Acids Res. (2013) 41:933–42. 10.1093/nar/gks1038 23221633 PMC3553971

[92] Bornstein S White R Malkoski S Oka M Han G Cleaver T. Smad4 loss in mice causes spontaneous head and neck cancer with increased genomic instability and inflammation. J Clin Invest. (2009) 119:3408–19. 10.1172/JCI38854 19841536 PMC2769185

[93] Moskwa P Buffa FM Pan Y Panchakshari R Gottipati P Muschel RJ. miR-182-mediated downregulation of BRCA1 impacts DNA repair and sensitivity to PARP inhibitors. Mol Cell. (2011) 41:210–20. 10.1016/j.molcel.2010.12.005 21195000 PMC3249932

[94] Martinez-Ruiz H Illa-Bochaca I Omene C Hanniford D Liu Q Hernando E. A TGFbeta-miR-182-BRCA1 axis controls the mammary differentiation hierarchy. Sci Signal. (2016) 9:ra118. 10.1126/scisignal.aaf5402 27923913 PMC5619986

[95] Brown JA Yonekubo Y Hanson N Sastre-Perona A Basin A Rytlewski JA. TGF-β-induced quiescence mediates chemoresistance of tumor-propagating cells in squamous cell carcinoma. Cell Stem Cell. (2017) 21:650–64.e658. 10.1016/j.stem.2017.10.001 29100014 PMC5778452

[96] Bouquet F Pal A Pilones KA Demaria S Hann B Akhurst RJ TGFb1 inhibition increases the radiosensitivity of breast cancer cells *in vitro* and promotes tumor control by radiation *in vivo*. Clin Cancer Res. (2011) 17:6754–65. 10.1158/1078-0432.CCR-11-0544 22028490 PMC3724539

[97] Hardee ME Marciscano AE Medina-Ramirez CM Zagzag D Narayana A Lonning SM. Resistance of glioblastoma-initiating cells to radiation mediated by the tumor microenvironment can be abolished by inhibiting transforming growth factor-beta. Cancer Res. (2012) 72:4119–29. 10.1158/0008-5472.CAN-12-0546 22693253 PMC3538149

[98] Du S Bouquet F Lo C-H Pellicciotta I Bolourchi S Parry R Attenuation of the DNA damage response by TGFβ inhibitors enhances radiation sensitivity of NSCLC cells *in vitro* and *in vivo*. Int J Radiat Oncol Biol Phys. (2014) 91:91–9. 10.1016/j.ijrobp.2014.09.026 25835621

[99] Weaver AN Cooper TS Rodriguez M Trummell HQ Bonner JA Rosenthal EL. DNA double strand break repair defect and sensitivity to poly ADP-ribose polymerase (PARP) inhibition in human papillomavirus 16-positive head and neck squamous cell carcinoma. Oncotarget. (2015) 6:26995–7007. 10.18632/oncotarget.4863 26336991 PMC4694969

[100] Mirghani H Amen F Tao Y Deutsch E Levy A. Increased radiosensitivity of HPV-positive head and neck cancers: molecular basis and therapeutic perspectives. Cancer Treat Rev. (2015) 41:844–52. 10.1016/j.ctrv.2015.10.001 26476574

[101] Ziemann F Arenz A Preising S Wittekindt C Klussmann JP Engenhart-Cabillic R. Increased sensitivity of HPV-positive head and neck cancer cell lines to x-irradiation ± Cisplatin due to decreased expression of E6 and E7 oncoproteins and enhanced apoptosis. Am J Cancer Res. (2015) 5:1017–31. 26045983 PMC4449432

[102] Xu Q Wang S Xi L Wu S Chen G Zhao Y. Effects of human papillomavirus type 16 E7 protein on the growth of cervical carcinoma cells and immuno-escape through the TGF-beta1 signaling pathway. Gynecol Oncol. (2006) 101:132–9. 10.1016/j.ygyno.2005.09.051 16269171

[103] Perez-Plasencia C Vazquez-Ortiz G Lopez-Romero R Pina-Sanchez P Moreno J Salcedo M. Genome wide expression analysis in HPV16 cervical cancer: identification of altered metabolic pathways. Infect Agent Cancer. (2007) 2:16. 10.1186/1750-9378-2-16 17822553 PMC2034543

[104] Li ML Greenberg RA. Links between genome integrity and BRCA1 tumor suppression. Trends Biochem Sci. (2012) 37:418–24. 10.1016/j.tibs.2012.06.007 22836122 PMC3459146

[105] Deng Y Deng H Liu J Han G Malkoski S Liu B. Transcriptional down-regulation of Brca1 and E-cadherin by CtBP1 in breast cancer. Mol Carcinog. (2012) 51:500–7. 10.1002/mc.20813 21681822 PMC3177013

[106] Bennardo N Cheng A Huang N Stark JM. Alternative-NHEJ is a mechanistically distinct pathway of mammalian chromosome break repair. PLoS Genet. (2008) 4:e1000110. 10.1371/journal.pgen.1000110 18584027 PMC2430616

[107] Bjorkman A Qvist P Du L Bartish M Zaravinos A Georgiou K. Aberrant recombination and repair during immunoglobulin class switching in BRCA1-deficient human B cells. Proc Natl Acad Sci USA. (2015) 112:2157–62. 10.1073/pnas.1418947112 25646469 PMC4343177

[108] Korkut A Zaidi S Kanchi RS Rao S Gough NR Schultz A. A pan-cancer analysis reveals high-frequency genetic alterations in mediators of signaling by the TGF-β superfamily. Cell Syst. (2018) 7:422–37.e7. 10.1016/j.cels.2018.08.010 30268436 PMC6370347

[109] Kanamoto T Hellman U Heldin CH Souchelnytskyi S. Functional proteomics of transforming growth factor-β1-stimulated Mv1Lu epithelial cells: Rad51 as a target of TGFβ1-dependent regulation of DNA repair. EMBO J. (2002) 21:1219–30. 10.1093/emboj/21.5.1219 11867550 PMC125881

[110] Liu L Zhou W Cheng CT Ren X Somlo G Fong MY. TGFβ induces “BRCAness” and sensitivity to PARP inhibition in breast cancer by regulating DNA-repair genes. Mol Cancer Res. (2014) 12:1597–609. 10.1158/1541-7786.MCR-14-0201 25103497 PMC4233161

[111] Zhang H Kozono DE O'connor KW Vidal-Cardenas S Rousseau A Hamilton A. TGF-β inhibition rescues hematopoietic stem cell defects and bone marrow failure in fanconi anemia. Cell Stem Cell. (2016) 18:668–81. 10.1016/j.stem.2016.03.002 27053300 PMC4860147

[112] Pal D Pertot A Shirole NH Yao Z Anaparthy N Garvin T. TGF-beta reduces DNA ds-break repair mechanisms to heighten genetic diversity and adaptability of CD44^+^/CD24^−^ cancer cells. Elife. (2017) 6:21615. 10.7554/eLife.21615 28092266 PMC5345931

